# Prescribing trends of SGLT2 inhibitors among HFrEF and HFpEF patients with and without T2DM, 2013–2021

**DOI:** 10.1186/s12872-024-03961-5

**Published:** 2024-05-30

**Authors:** Jimmy Gonzalez, Chintan V. Dave

**Affiliations:** 1 Department of Pharmacy Practice and Administration, Ernest Mario School of Pharmacy, Rutgers University, Piscataway, NJ USA; 2 Department of Pharmacy, Jersey Shore University Medical Center, Neptune, NJ USA; 3 Center for Pharmacoepidemiology and Treatment Science, Institute for Health, Health Care Policy and Aging Research, Rutgers University, New Brunswick, NJ USA

**Keywords:** Sodium-glucose cotransporter-2 inhibitors, Heart failure with reduced ejection fraction, Heart failure with preserved ejection fraction, Prescribing trend

## Abstract

**Background:**

Sodium-glucose cotransporter-2 inhibitors (SGLT2i) are recommended for treatment of heart failure (HF), regardless of type 2 diabetes (T2DM) status. However, limited data exist on SGLT2i prescribing in HF patients without T2DM or across HF subtypes.

**Methods:**

This was a serial, cross-sectional study of US MarketScan commercial and Medicare claims (2013–2021). Prevalence of SGLT2i was calculated by calendar year among HFrEF and HFpEF patients and stratified by T2DM status.

**Results:**

Among 218,066 HFrEF patients [mean (SD): 54.9 (8.92) years; 66.4% male], the prevalence of SGLT2i use increased from 0.3 to 18.6%, while among 150,437 HFpEF patients [56.5 (7.77) years; 47.6% male], it rose from 0.5 to 9.9%. These increases were driven by the subgroup with comorbid T2DM. SGLT2i prevalence use ratios among patients with T2DM compared to those without decreased from > 100 in 2018 to 3.8 in 2021 among HFrEF patients, and from 83.1 in 2018 to 17.5 in 2021, coinciding with the publication of landmark trials and corresponding changes in clinical guidelines.

**Conclusions:**

SGLT2i use rose rapidly following changes in guidelines but remained low among those without T2DM. By the end of the study, approximately 1 in 3 HFrEF and 1 in 5 HFpEF patients with T2DM were using an SGLT2i, compared to only 1 in 11 HFrEF and 1 in 85 HFpEF patients without T2DM. Future work identifying barriers with the uptake of GDMT, including SGLT2i, among HF patients is needed.

**Supplementary Information:**

The online version contains supplementary material available at 10.1186/s12872-024-03961-5.

## Introduction

Sodium glucose co-transporter 2 inhibitors (SGLT2i) are a newer drug class originally approved for type 2 diabetes mellitus (T2DM). Initial cardiovascular outcomes trials conducted in T2DM patients showed that empagliflozin [1], canagliflozin [2], and dapagliflozin [3] reduced the incidence of hospitalization for heart failure (HHF), spurring dedicated trials in patients with heart failure with reduced ejection fraction (HFrEF) [4, 5], and subsequently, patients with heart failure with preserved ejection fraction (HFpEF) [6, 7]. Data from both clinical trials and real world studies support the use of SGLT2i in patients with HFrEF and HFpEF [8, 9].

Consequently, package inserts from the Food & Drug Administration for select SGLT2i – namely dapagliflozin and empagliflozin – now include new indications highlighting the reduced risk of HHF among patients with heart failure regardless of comorbid T2DM diagnosis. Notably, canagliflozin possesses this indication only with comorbid T2DM and ertugliflozin lacks the indication completely. Further, the American College of Cardiology (ACC) Expert Consensus decision pathway for HF was updated in 2021 to include SGLT2i, and the most recent joint American Heart Association, Heart Failure Society of America, and ACC guidelines recommend the first line use of SGLT2i for HF patients regardless of T2DM status [10, 11].

Despite the recent publication of landmark trials, and the corresponding changes in drug labels and clinical guidelines, there is lack of data examining the use of SGLT2i among HF patients with and without T2DM. While SGLT2i have unique characteristics that favor early adoption in HF, such as convenient once-daily dosing and good tolerability, concerns linger regarding their uptake. First, prior studies have revealed the slow and variable uptake of newer HF therapies that have demonstrated similar reductions in morbidity and mortality [12]. Second, there may be greater reluctance to prescribe these therapies to HF patients without comorbid T2DM due to them being primarily glucose lowering therapies, representing a departure from other conventional HF treatments.

The utility of prior studies examining similar topics are limited by their small sample sizes or single center settings [13], focus on T2DM populations [14, 15], lack of differentiation between HFrEF and HFpEF, and absence of data on uptake of individual agents within the class. More importantly, limited research has specifically examined SGLT2i use in HF patients without T2DM. Accordingly, the objective of this study was to evaluate prescribing trends of SGLT2i among commercially insured patients with HF in the US between 2013 and 2021, and to quantify the variations in uptake by T2DM status.

## Methods

The study did not meet the regulatory definition of human subjects research provided in 45 CFR 46.102 and thus was approved by the Rutgers University IRB as non-human subject research; appropriate data use agreements were in place.

### Data source

Study subjects were drawn from the MarketScan Commercial and Medicare Database from 2013 to 2021. MarketScan is an administrative claims database comprised of individual-level, deidentified healthcare data in the US. Data elements of interest included patient demographics (e.g., age, sex, ), medical and pharmacy enrollment status, inpatient and outpatient medical encounters (International Classification of Disease [ICD], Ninth and Tenth Revisions), and outpatient pharmacy prescription data (drug name and date dispensed).

### Study population and prevalence of SGLT2i use

We conducted a serial, cross-sectional study, stratifying analyses by calendar year. To be included, patients were required to maintain continuous eligibility for pharmacy and medical benefits throughout a specific calendar year. For each year, two mutually exclusive cohorts representing patients with HFrEF (Cohort 1; 428.2x (ICD-9) or I50.2x (ICD-10)) and HFpEF (Cohort 2; 428.3x (ICD-9) or I50.3x (ICD-10)) were created based on corresponding ICD claims in either one inpatient or two outpatient encounters at least 30-days apart within that year. We excluded patients aged < 18 years, those with diagnosis codes for chronic kidney disease (CKD) stage 5 [(585.5, 585.6 (ICD-9) or N18.5, N18.6 (ICD-10))] or dialysis (Z49.0 (ICD-10)) – representing enrollees with eGFR < 15, or the presence of both HFrEF and HFpEF codes. T2DM was identified using ICD claims codes [(250.X (except 250.x1 and 250.x3) (ICD-9)) or (E11 (ICD-10))]. CKD stage 4 patients were included in our study due to increasing evidence of use in this population for renoprotective effects.[16] Positive predictive values of 90% and 92% were noted in a previous chart validation of the HFrEF and HFpEF ICD-10 diagnosis codes algorithm [17].

To estimate the prevalence of use of SGLT2i, we enumerated the proportion of patients using these agents (numerator) among those with HFrEF (Cohort 1 denominator) and HFpEF (Cohort 2 denominator) for each calendar year. Patients were allowed to contribute more than one episode (over different calendar years) as long as the inclusion and exclusion criteria were met for that year.

### Statistical analyses

All analyses were performed using SAS 9.4 (SAS Institute Inc, Cary, NC). In addition to ascertaining information on heart failure status and SGLT2i use, we also assessed information on relevant baseline clinical characteristics including sociodemographic variables (e.g., age, biological sex), and medical conditions (e.g., myocardial infarction, stroke, CKD, and T2DM).

Plots were generated to examine secular trends in the prevalence of SGLT2i use among HFrEF and HFpEF patients, and were further stratified based on T2DM status. Publication of landmark trials and changes in drug labelling and guidelines were indicated on the plots to identify potential inflection points. Additionally, comparison of the annual individual agents within the class were examined. For all analyses, we conducted Cochrane-Armitage tests to assess for presence of a trend and reported the corresponding p-values.

Finally, we conducted a sensitivity analysis where patients were stratified into older (≥ 65 years) and younger (< 65 years) age groups to explore variations in SGLT2i prescribing patterns among different age cohorts of HF patients given possible differences in baseline population sourced from MarketScan Commercial vs. MarketScan Medicare.

## Results

Initially, we identified a total of 790,167 and 772,773 distinct HF encounters among adult patients (aged > 18 years) within the HFrEF and HFpEF cohorts, respectively; after applying the inclusion and exclusion criteria, we identified 218,066 HFrEF and 150,437 HFpEF episodes between 2013 and 2021 (Table 1, see Supplemental Fig. 1 for the CONSORT flow diagram). In the HFrEF cohort, the most common guideline-recommended medication classes prescribed were beta blockers (84.7%) followed by loop diuretics (55.3%). The prevalence of comorbidities and selected heart failure medications varied slightly between the HFrEF and HFpEF cohorts; for instance, HFrEF patients were more likely to be diagnosed with MI (13.4% vs. 6.2%) and less likely to have CKD (11.0% vs. 13.0%) compared their counterparts in the HFpEF group. Additional breakdown by T2DM status and age group is available in Appendix Tables 1 and 2, respectively.

**Table 1:** Baseline characteristics of eligible episodes stratified by heart failure subtype in MarketScan Commercial and Medicare data from 2013–2021

	HFrEF (*n* = 218,066)	HFpEF (*n* = 150,437)
**Sociodemographics**		
Age, mean (SD)	54.9 (8.92)	56.5 (7.7)
Male sex	144,882 (66.4)	71,639 (47.6)
**Calendar year**		
2013	17,837 (8.2)	12,523 (8.3)
2014	23,503 (10.8)	15,942 (10.6)
2015	21,272 (9.8)	14,064 (9.4)
2016	26,451 (12.1)	17,162 (11.4)
2017	25,546 (11.7)	17,183 (11.4)
2018	26,069 (12)	18,347 (12.2)
2019	25,697 (11.8)	18,582 (12.4)
2020	25,775 (11.8)	18,078 (12)
2021	25,916 (11.9)	18,556 (12.3)
**Comorbidities**		
MI	29,204 (13.4)	9,350 (6.2)
CKD	23,967 (11.0)	19,546 (13.0)
CVA	20,860 (9.6)	16,451 (10.9)
T2DM	83,835 (38.4)	65,735 (43.7)
**Medications**		
ACEi	103,948 (47.7)	49,392 (32.8)
ARB	54,758 (25.1)	42,741 (28.4)
ARNI	33,291 (15.3)	1,157 (0.8)
Aldosterone antagonist	76,702 (35.2)	23,274 (15.5)
Beta blocker	184,716 (84.7)	91,956 (61.1)
Digoxin	22,818 (10.5)	4,573 (3.0)
Hydralazine/ISDN	1,690 (0.8)	343 (0.2)
Ivabradine	2,146 (1.0)	180 (0.1)
Loop diuretic	120,556 (55.3)	76,210 (50.7)
SGLT2i	12,889 (5.9)	7,134 (4.7)

**Abbreviations**: ACEi: Angiotensin converting enzyme inhibitor; ARB: Angiotensin II receptor blocker; ARNI: angiotensin receptor/neprilysin inhibitor; CKD: chronic kidney disease; CVA: cerebrovascular accident; HFrEF: Heart failure with reduced ejection fraction; HFpEF: Heart failure with preserved ejection fraction; ISDN: Isosorbide dinitrate; MI: Myocardial infarction; SGLT2i: Sodium-glucose cotransporter-2 inhibitor; SD: Standard deviation

### Overall trends in patients by HFrEF or HFpEF status

**Fig. 1:**
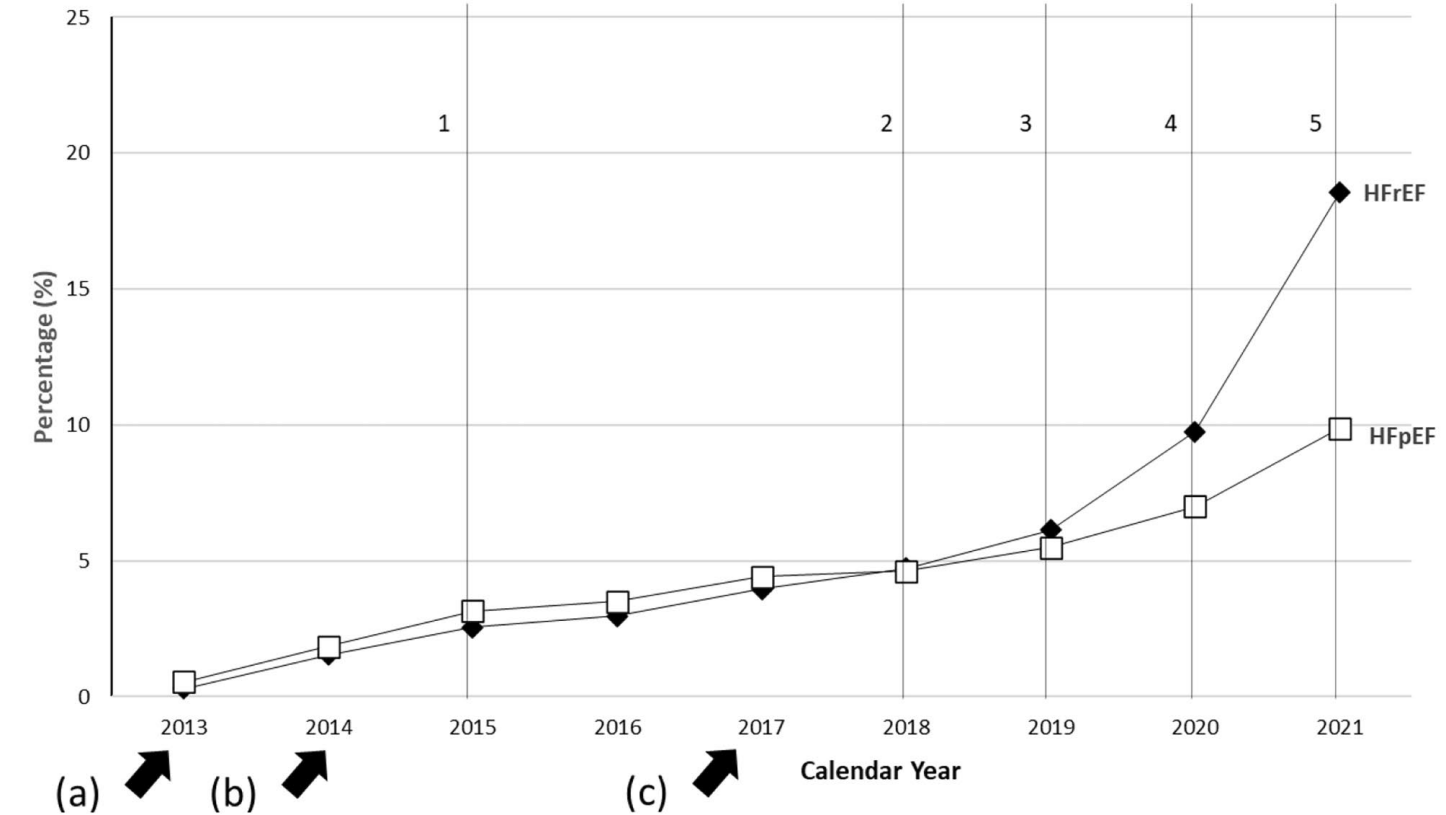
Comparative trends of SGLT2i prescribing prevalence among the HFrEF and HFpEF cohorts. Relative prevalence of all SGLT2i prescribing (canagliflozin, dapagliflozin, empagliflozin, ertugliflozin) within the HFrEF and HFpEF cohorts identified in MarketScan commercial and Medicare claims between January 2013 and December 2021. *a)* FDA approval of canagliflozin, *b)* FDA approval of both dapagliflozin in Q1 and empagliflozin in Q3, *c*) FDA approval of ertugliflozin. 1: EMPA-REG is published demonstrating CVD benefits in patients with T2DM. 2: American Diabetes Association guidelines recommends SGLT2i as a second-line therapy for patients with T2DM and CVD. 3: DAPA-HF demonstrates dapagliflozin reduces risk of worsening HF and CV mortality regardless of T2DM status. 4: EMPEROR-REDUCED finds decreased CV death and HHF among HFrEF patients treated with empagliflozin; dapagliflozin receives FDA indication for HF. 5: Update to ACC heart failure decision algorithm promotes dapagliflozin and empagliflozin as a first-line component of guideline-directed medical therapy

**Fig. 2:**
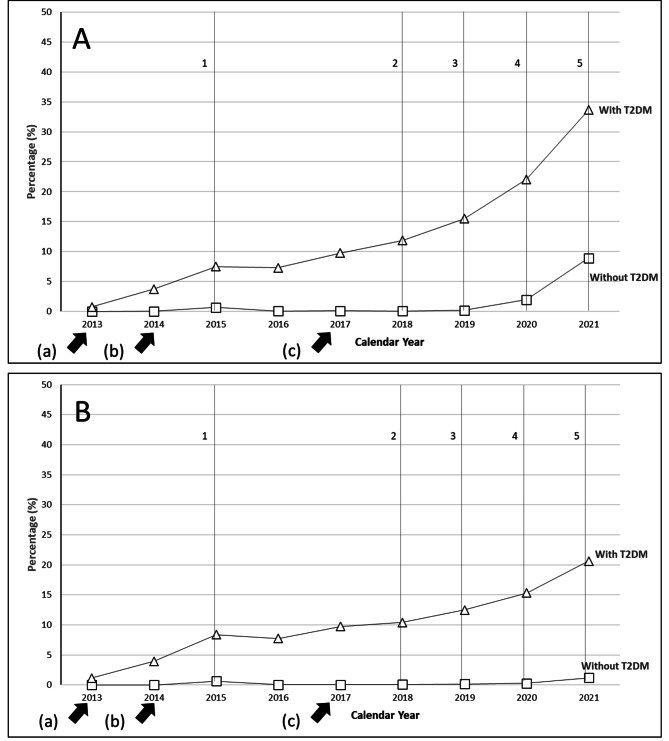
Trends in prescribing patterns of SGLT2i medications among patients with HFrEF and HFpEF stratified by T2DM. Relative prevalence of all SGLT2i prescribing (canagliflozin, dapagliflozin, empagliflozin, ertugliflozin) within the HFrEF cohort **(A)** and HFpEF cohort **(B)** identified in MarketScan commercial and Medicare claims between January 2013 and December 2021. *a)* FDA approval of canagliflozin, *b)* FDA approval of both dapagliflozin in Q1 and empagliflozin in Q3, *c*) FDA approval of ertugliflozin. 1: EMPA-REG is published demonstrating CVD benefits in patients with T2DM. 2: American Diabetes Association guidelines recommends SGLT2i as a second-line therapy for patients with T2DM and CVD. 3: DAPA-HF demonstrates dapagliflozin reduces risk of worsening HF and CV mortality regardless of T2DM status. 4: Dapagliflozin receives FDA indication for HF. 5: EMPEROR-PRESERVED finds CV benefits with empagliflozin in HFpEF patients; update to ACC heart failure decision algorithm promotes dapagliflozin and empagliflozin as a first-line component of guideline-directed medical therapy

Throughout the study period, SGLT2i use was higher in the HFrEF cohort compared to the HFpEF cohort, with SGLT2i prescribing rates in the HFrEF cohort overtaking those in the HFpEF cohort rapidly starting in 2020 (Fig. 1). Among the overall cohort of patients with HFrEF, the prevalence of use for SGLT2i increased by 18.3%, from 0.3% in 2013 to 18.6% in 2021 (p-value for trend: <0.001), with the highest increases seen after 2020 following the publication of the EMPEROR-REDUCED trial (Fig. 2A). Among the overall cohort of patients with HFpEF, the prevalence of use of SGLT2i increased by 9.4%, from 0.5% in 2013 to 9.9% in 2021 (*p* < 0.001) (Fig. 2B).

### Secular trends in patients with or without T2DM

Changes in prescribing trends were more apparent within each HF subgroup with a concomitant diagnosis of T2DM than those without. For instance, by 2019, 15.5% of patients with HFrEF and T2DM were on an SGLT2i compared to < 1% of patients without T2DM. Additionally, there was a rapid increase in the use of SGLT2i in patients with T2DM (15.5% [2019] to 33.6% [2021]) and without T2DM (0.2% [2019] to 8.9% [2021]). However, the trend observed among the HFrEF-only stratum failed to reach significance (*p* = 0.08).

While the overall prevalence of SGLT2i use grew between 2013 and 2021 among the HFpEF cohort, this change was driven primarily by patients with T2DM (*p* < 0.001). By contrast, the trend was not significant among those without T2DM (*p* = 0.22) with only a 0.3% increase in prescribing prevalence apparent from 2013 to 2020. A large relative increase in SGLT2i prescribing was noted in patients with T2DM (15.3% [2020] to 20.6% [2021]) and a modest increase in patients without T2DM (0.3% [2020] to 1.2% [2021]), after the publication of the EMPEROR-PRESERVED trial in 2020.

Prior to 2019 and before the publication of EMPEROR-REDUCED, the prevalence of SGLT2i ratio in HFrEF patients with T2DM compared to those without T2DM was > 100. Thereafter, this ratio decreased from 86.0 (15.5% vs. 0.2%) in 2019, to 11.2 in 2020, and eventually reaching 3.8 (33.6% vs. 8.9%) by the end of the study period in 2021. Similarly, the prevalence of use ratio among patients with and without T2DM was also considerably higher in the HFpEF group by the end of the study period, albeit decreasing from 83.1 (12.5% vs. 0.2%) to 17.5 (20.6% vs. 1.2%).

### Individual agents

**Fig. 3:**
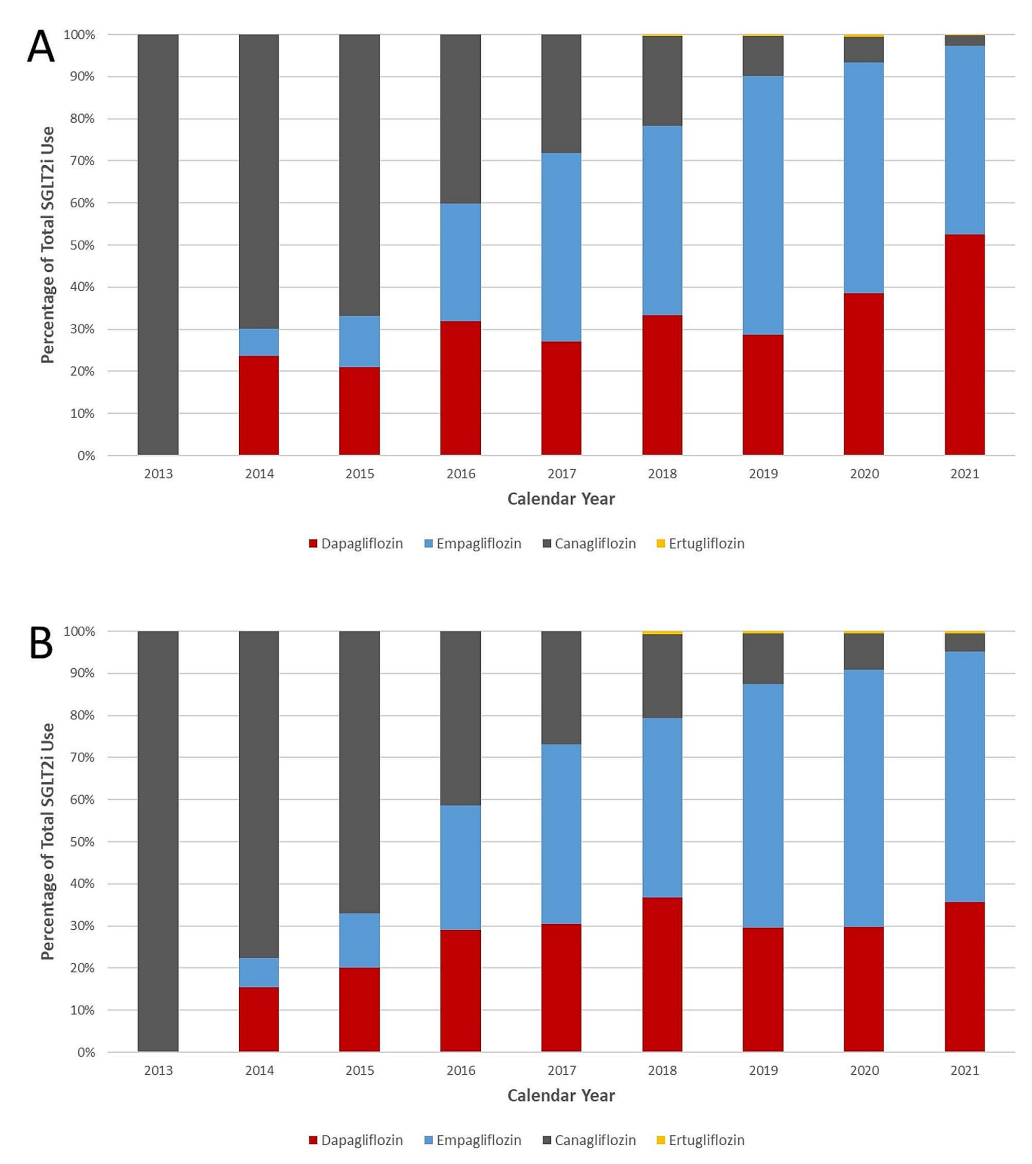
Relative SGLT2i prescribing prevalence stratified by HF status. The relative percentages of canagliflozin, dapagliflozin, empagliflozin, and ertugliflozin market share are depicted in the **(A)** HFrEF and **(B)** HFpEF cohorts regardless of T2DM status

Large changes in market share were observed among canagliflozin, dapagliflozin, empagliflozin, and ertugliflozin (Fig. 3A-B). Canagliflozin market share dropped precipitously from the only marketed SGLT2i (100% [2013]) to a significantly reduced level (2.5% [2021]). Dapagliflozin maintained a relatively constant prescription share of 21.6–39.5% in the HFrEF cohort until realizing a large gain to 53.8% in 2021. Empagliflozin prescribing grew noticeably in 2017 (47.4%) and 2019 (63.1%). Similar trends were observed within the HFpEF cohort with a slightly higher prevalence of empagliflozin over dapagliflozin.

#### Sensitivity analysis

**Fig. 4:**
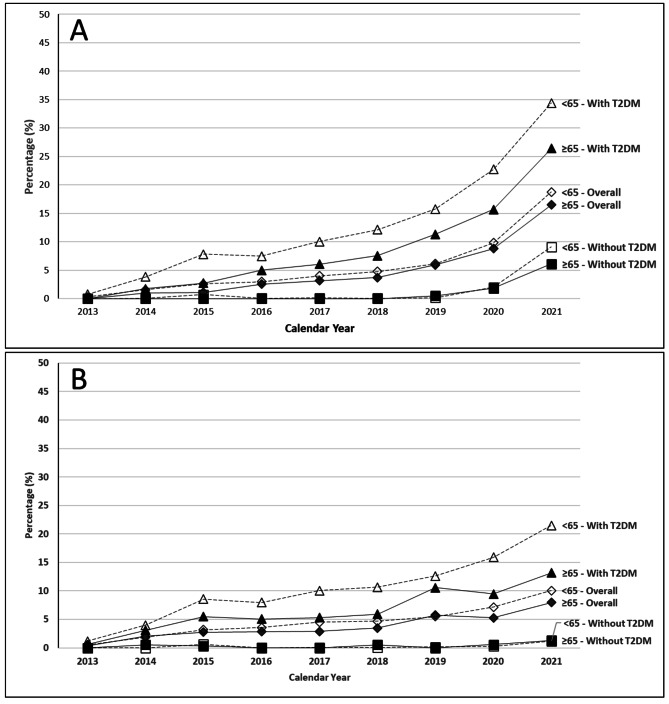
SGLT2i trends analysis stratified by age ≥ 65 years or < 65 years and T2DM status. Relative prevalence of all SGLT2i prescribing (canagliflozin, dapagliflozin, empagliflozin, ertugliflozin) within the HFrEF cohort identified in MarketScan commercial and Medicare claims between January 2013 and December 2021 within the **(A)** HFrEF cohort and **(B)** HFpEF cohort

SGLT2i prescribing trends were mostly similar when stratified by age 65 and greater and age less than 65, though greater utilization was noted in patients with T2DM and in the younger age stratum (Fig. 4A-B).

## Discussion

In this nationwide study of commercially insured patients, we found that the use of SGLT2i increased steadily among patients with HFrEF and HFpEF over the study period from 2013 to 2019, and rapidly thereafter, coinciding with the publication of major CVOTs and corresponding guideline updates. We also identified variations in the uptake of SGLT2i, with a more rapid uptake observed in patients with T2DM. For instance, by the end of the study period in 2021, approximately 1 in 3 HFrEF patients with T2DM and 1 in 11 HFrEF patients without T2DM were prescribed an SGLT2i. Similarly, 1 in 5 HFpEF patients with T2DM and 1 in 85 HFpEF patients without T2DM were prescribed these medications. These results suggest a large pool of potentially eligible HF patients, especially those without concomitant T2DM, were not being prescribed these guideline-recommended medical treatments. Thus, medication therapy for many HF patients could be optimized to realize the major cardiovascular benefits and reductions in hospitalization for heart failure imparted by SGLT2i.

Limited data are available regarding national trends in the utilization of SGLT2i across different subtypes of HF and among HF patients without T2DM. However, several studies have explored SGLT2i utilization in T2DM populations, revealing lower rates of utilization among patients with cardiorenal conditions [14, 16, 18]. One drug utilization review saw the overall proportion of patients prescribed SGLT2i with CV disease or HF only increased by 3.4% from 2013 to 2018 with the proportion prescribed by cardiologists marginally increased from 1.4 to 3.6% [14]. Another study using UK data found SGLT2i were used with slightly lower frequency in patients with T2DM and CVD (9.8%) compared to those without CVD (13.8%) [15]. Specific to heart failure, an electronic health records study found low SGLT2i uptake between 2013 and 2019 [13]. Variable and low prescribing rates (∼ 20%) were also noted in an analysis of HFrEF patients hospitalized and enrolled into the Get With The Guidelines-Heart Failure registry [19]. Notably, average age and GDMT medication prevalence were higher in this registry report than found in our analysis reflecting a difference in the source populations between studies due to the commercial claims data source. A stratified analysis by age over and under 65 years was included to address this concern (Appendix Table 2), though SGLT2i use among older adults was still lower than the younger cohort across all HF subgroups (Fig. 4A-B). Interestingly, dapagliflozin prescription share among the HFrEF cohort increased in 2020 and 2021, which followed the pivotal DAPA-HF trial, though no increase was noted within the HFpEF cohort, potentially due to predating its pivotal DELIVER trial which was published in 2022 (Fig. 4A-B) [5, 20].

Our study highlighted a decreasing, albeit persistent, disparity in SGLT2i utilization between HF patients with and without comorbid T2DM. The dual diagnosis of T2DM and HF is associated with higher healthcare burden, polypharmacy and overall clinical complexity [21, 22], which may pose challenges in initiating additional therapies. Nevertheless, our findings indicate that patients with T2DM were more likely to initiate SGLT2i treatment. This discrepancy may be attributed to the historical perception of SGLT2i being primarily antidiabetic medications rather than modulators of cardiovascular disease risk. Nevertheless, limited data exist detailing secular trends of insurance plan coverage for SGLT2i for any indication. Recent studies report vastly different coverage for individual SGLT2i among Medicare Part D beneficiaries and commercial third-party beneficiaries [23]. Furthermore, only 50–60% of commercial plan enrollees had coverage for SGLT2i without prior authorization or step therapy requirements [24]. Regardless, HF patients without T2DM stand to benefit greatly from the use of these therapies, and their underutilization in this population highlights the need to identify and address the barriers associated with their reduced uptake.

Additionally, while this study is unable to attribute the low use of SGLT2i to any particular cause, the following factors may play a role. First, prescribing inertia has been postulated to explain low adherence to HF guidelines, but a recent analysis found physiologic factors (e.g., age, history of CVA) instead were significantly associated with therapy gaps [25]. Further, SGLT2i CVOTs and supporting guideline updates are relatively recent in publication and diffusion of new practices may be limited to prescriptive innovators. Second, restrictive formularies (in the initial study years) among third party commercial insurers in the US may have also contributed to their low use. Third, the unique adverse effect profile of SGLT2i, including potential concerns for diabetic ketoacidosis and urogenital infections [26–30], may contribute to provider and patient hesitancy in using these therapies. Lastly, other patient level factors, which include lower baseline BMI or frailty may have also contributed to the hesitancy in prescribing SGLT2i in certain patient populations [31].

Currently, limited treatment options exist for HFpEF. Traditionally, antihypertensives have been used to reduce afterload and prevent worsening of left ventricular hypertrophy. Prior to the publication of EMPEROR-PRESERVED, limited data suggested aldosterone antagonists and angiotensin receptor blockers may reduce HHF in this population; however, these benefits were less robust and not inclusive of mortality benefit as seen with SGLT2i [11]. Given that these medications represent one of the only effective treatment modalities that reduce HHF and CVD mortality in HFpEF patients, we anticipate the use of these therapies to grow over time.

### Strengths

This study features many strengths. Foremost, this is the first analysis of SGLT2i prescribing trends among a HF population following the publication of updated guidelines supporting use of SGLT2i among patients with HFrEF and HFpEF. The use of the MarketScan claims database – which includes data on millions of adult patients in the US – provides a real-world perspective on secular prescribing trends. Furthermore, the investigators include very recent data (up to 2021) which allowed for a comprehensive evaluation with current public health implications. Finally, examination of SGLT2i trends among HF patients without T2DM is a key study strength, as it provides valuable insights into the variation in utilization patterns by this key subgroup.

### Limitations

However, the study is limited in the following ways. First, study results are generalizable to patients with commercial insurance (approximately 55% of the US population) and Medicare supplemental insurance within the MarketScan database. Second, the observed prescribing trends may reflect variation in formulary coverage of SGLT2i (e.g., prior authorizations, step therapy) and shared patient costs, especially in the initial study years and for patients without a diagnosis of T2DM [24, 25]. Third, as our study ended in 2021, we were unable to study trends attributable to the publication of newer CVOTs [7], nor fully account for the impact of the COVID-19 pandemic.

The decision to exclude patients with concurrent HFrEF and HFpEF codes was done to prioritize positive predictive values but may limit generalizability. Nonetheless, our past research demonstrated that codes for combined HFpEF and HFrEF had limited accuracy in identifying patients with HFrEF (PPV = 45%) or HFpEF (PPV = 21%) [17]. However, we cannot understate that exclusion of patients with both codes also excludes patients with gradually improving systolic function (improved LVEF) or gradually worsening function (transitioning from HFpEF to HFrEF). Like other commercial claims databases such as Optum, Anthem, and TriNetX, MarketScan does not contain information on race or ethnicity [32], as private insurance companies do not routinely collect – or in certain states, are explicitly prohibited from collecting – race and ethnicity data. Similarly, administrative claims do not provide details on individual prescribing decisions, patient refusals, or medication discontinuations due to adverse effects. Finally, HF classification was achieved using diagnostic coding rather than by ejection fraction which were unavailable in these data, though high positive predictive values for this technique have been reported [17]. Similar utilization of beta blockers (85%) and other treatments among patients in our study, in the recent guideline registry analysis for HFrEF (89%) [19], and the EMPEROR-PRESERVED trial (81%) [6], suggests our sample was derived from the same background populations. Regardless, administrative claims definitions of HF are susceptible to bias due to heterogeneity in clinical presentation, the evolving nomenclature of HF, and subtle differences between systolic/diastolic dysfunction in relation to pathophysiology vs. clinical presentation [33, 34].

## Conclusion

This study found increased SGLT2i prescribing prevalence over time among HF patients, regardless of T2DM status, marked by dramatic upticks in prescribing at inflection points coinciding with publication of major clinical trials and guidelines. Stratification by T2DM status revealed most SGLT2i prescribing in this commercial claims cohort was driven by use among patients with T2DM, and very few patients without comorbid T2DM (< 10%) were initiated on SGLT2i despite new literature and guideline recommendations. While the relative increases in proportional market share of empagliflozin and dapagliflozin support diffusion of updated guideline recommendations, the low absolute uptake represents a significant area for pharmacotherapy optimization for reducing HF morbidity and mortality.

### Electronic supplementary material

Below is the link to the electronic supplementary material.

Supplementary Material 1

## Data Availability

The data that support the findings of this study are available from IBM MarketScan and Medicare but restrictions apply to the availability of these data, which were used under license for the current study, and so are not publicly available.
