# Supplementary material for: Prescribing trends of SGLT2 inhibitors among HFrEF and HFpEF patients with and without T2DM, 2013–2021
Source: BMC Cardiovasc Disord. 2024 May 30;24:285. doi: 10.1186/s12872-024-03961-5 (PMC11137883; doi:10.1186/s12872-024-03961-5)
Supplement: Supplementary file 1 — Supplementary Material 1 [file 12872_2024_3961_MOESM1_ESM.docx]

**SUPPLEMENTARY APPENDIX**

**Supplemental Figure 1.** CONSORT flow diagram

**Appendix Table 1:** Baseline patient characteristics stratified by diabetes status among patients with heart failure with reduced and preserved ejection fraction

**Appendix Table 2:** Sensitivity analysis of cohort stratified by heart failure subtype and age group

**Supplemental Figure 1.** CONSORT flow diagram


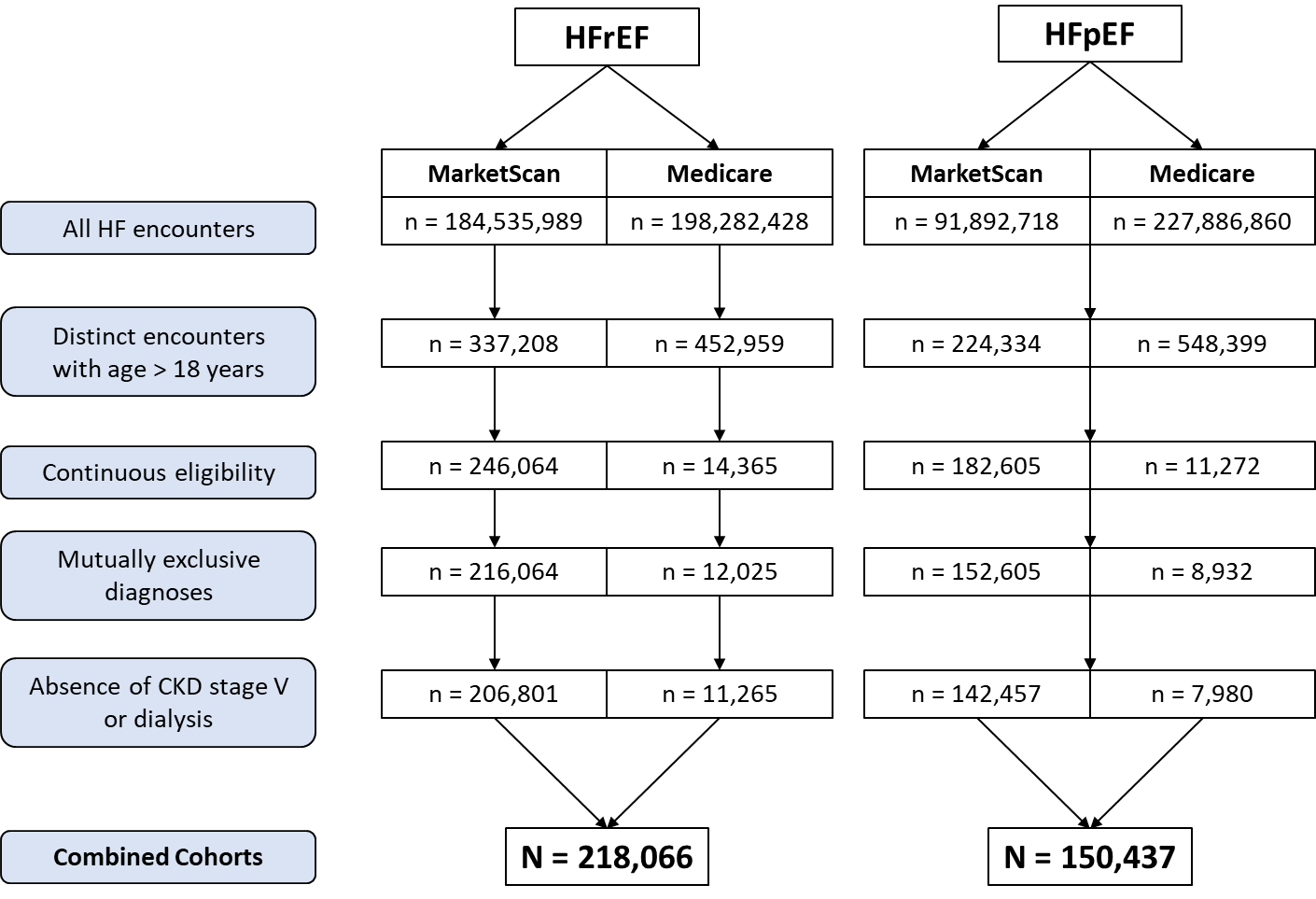


| **Appendix Table 1.** Baseline patient characteristics among patients with heart failure with reduced and preserved ejection fraction stratified by diabetes status | | | | |
| --- | --- | --- | --- | --- |
| **Characteristic, n (%)** | **HFrEF**  **(n=218,066)** | | **HFpEF**  **(n=150,437)** | |
|  | Without T2DM  (n=134,231) | With T2DM  (n=83,835) | Without T2DM (n=94,204) | With T2DM  (n=76,812) |
| **Sociodemographics** |  |  |  |  |
| Age, mean (SD) | 53.9 (9.57) | 56.6 (7.49) | 55.6 (8.36) | 57.6 (6.70) |
| Male sex | 87,537 (65.2) | 57,345 (68.4) | 38,808 (45.8) | 32,831 (49.9) |
| **Calendar year** |  |  |  |  |
| 2013 | 10,650 (7.9) | 7,187 (8.6) | 6,773 (8.0) | 5,750 (8.8) |
| 2014 | 13,926 (10.4) | 9,577 (11.4) | 8,546 (10.1) | 7,396 (11.3) |
| 2015 | 15,434 (11.5) | 5,838 (7.0) | 9,481 (11.2) | 4,583 (7.0) |
| 2016 | 15,851 (11.8) | 10,600 (12.6) | 9,431 (11.1) | 7,731 (11.8) |
| 2017 | 15,295 (11.4) | 10,251 (12.2) | 9,431 (11.1) | 7,752 (11.8) |
| 2018 | 15,777 (11.8) | 10,292 (12.3) | 10,280 (12.1) | 8,067 (12.3) |
| 2019 | 15,704 (11.7) | 9,993 (11.9) | 10,528 (12.4) | 8,054 (12.3) |
| 2020 | 15,804 (11.8) | 9,971 (11.9) | 9,979 (11.8) | 8,099 (12.3) |
| 2021 | 15,790 (11.8) | 10,126 (12.1) | 10253 (12.1) | 8,303 (12.6) |
| **Comorbidities** |  |  |  |  |
| MI | 16,447 (12.3) | 12,757 (15.2) | 4,386 (5.2) | 4,964 (7.6) |
| CKD | 9,341 (7.0) | 14,626 (17.4) | 5,917 (7.0) | 13,629 (20.7) |
| CVA | 10,403 (7.8) | 10,457 (12.5) | 7,818 (9.2) | 8,633 (13.1) |
| **Medications** |  |  |  |  |
| ACEi | 64,245 (47.9) | 39,703 (47.4) | 23,980 (28.3) | 25,412 (38.7) |
| ARB | 30,936 (23.0) | 23,822 (28.4) | 20,470 (24.2) | 22,271 (33.9) |
| ARNI | 19,807 (14.8) | 13,484 (16.1) | 610 (0.7) | 547 (0.8) |
| Aldosterone antagonist | 45,669 (34.0) | 31,033 (37.0) | 11,325 (13.4) | 11,949 (18.2) |
| Beta blocker | 112,455 (83.8) | 72,261 (86.2) | 47,228 (55.8) | 44,728 (68.0) |
| Digoxin | 13,466 (10.0) | 9,352 (11.2) | 2,366 (2.8) | 2,207 (3.4) |
| Hydralazine/ISDN | 831 (0.6) | 859 (1.0) | 135 (0.2) | 208 (0.3) |
| Ivabradine | 1,084 (0.8) | 1,062 (1.3) | 97 (0.1) | 83 (0.1) |
| Loop diuretic | 66,422 (49.5) | 54,134 (64.6) | 36,256 (42.8) | 39,954 (60.8) |
| **SGLT2i** | 16,447 (12.3) | 12,757 (15.2) | 244 (0.3) | 6,890 (10.5) |
| Canagliflozin | 102 (0.1) | 1,915 (2.3) | 64 (0.1) | 1,513 (2.3) |
| Dapagliflozin | 1,311 (1.0) | 3,918 (4.7) | 102 (0.1) | 2,153 (3.3) |
| Empagliflozin | 500 (0.4) | 5,491 (6.5) | 79 (0.1) | 3,420 (5.2) |
| Ertugliflozin | 1 (0.0) | 34 (0.0) | 2 (0.0) | 28 (0.0) |
| **Abbreviations**: ACEi: Angiotensin converting enzyme inhibitor; ARB: Angiotensin II receptor blocker; ARNI: angiotensin receptor/neprilysin inhibitor; CKD: chronic kidney disease; CVA: cerebrovascular accident; HFrEF: Heart failure with reduced ejection fraction; HFpEF: Heart failure with preserved ejection fraction; ISDN: Isosorbide dinitrate; MI: Myocardial infarction; SGLT2i: Sodium-glucose cotransporter-2 inhibitor; SD: Standard deviation | | | | |

| **Appendix Table 2.** Sensitivity analysis of cohort stratified by heart failure subtype and age group | | | | |
| --- | --- | --- | --- | --- |
| **Characteristic, n (%)** | **HFrEF**  **(n=218,066)** | | **HFpEF**  **(n=150,437)** | |
|  | Age <65  (n=206,680) | Age ≥65  (n=11,386) | Age <65  (n=142,222) | Age ≥65  (n=8,215) |
| **Sociodemographics** |  |  |  |  |
| Age | 54.3 (8.68) | 66.9 (2.06) | 55.9 (7.53) | 66.8 (2.01) |
| Male sex | 137,078 (66.3) | 7,804 (68.5) | 67,805 (47.7) | 3,834 (46.7) |
| **Calendar year** |  |  |  |  |
| 2013 | 17,417 (8.4) | 420 (3.7) | 12,240 (8.6) | 283 (3.4) |
| 2014 | 22,767 (11) | 736 (6.5) | 15,495 (10.9) | 447 (5.4) |
| 2015 | 20,346 (9.8) | 926 (8.1) | 13,451 (9.5) | 613 (7.5) |
| 2016 | 24,837 (12.0) | 1,614 (14.2) | 16,104 (11.3) | 1,058 (12.9) |
| 2017 | 24,080 (11.7) | 1,466 (12.9) | 16,180 (11.4) | 1,003 (12.2) |
| 2018 | 24,831 (12.0) | 1,238 (10.9) | 17,540 (12.3) | 807 (9.8) |
| 2019 | 24,462 (11.8) | 1,235 (10.9) | 17,639 (12.4) | 943 (11.5) |
| 2020 | 23,903 (11.6) | 1,872 (16.4) | 16,599 (11.7) | 1,479 (18) |
| 2021 | 24,037 (11.6) | 1,879 (16.5) | 16,974 (11.9) | 1,582 (19.3) |
| **Comorbidities** |  |  |  |  |
| MI | 28,165 (13.6) | 1,039 (9.1) | 8,856 (6.2) | 494 (6.0) |
| CKD | 21,713 (10.5) | 2,254 (19.8) | 17,714 (12.5) | 1,832 (22.3) |
| CVA | 19,316 (9.3) | 1,544 (13.6) | 15,189 (10.7) | 1,262 (15.4) |
| T2DM | 78,133 (37.8) | 5,702 (50.1) | 61,254 (43.1) | 4,481 (54.5) |
| **Medications** |  |  |  |  |
| ACEi | 99,106 (48.0) | 4,842 (42.5) | 46,811 (32.9) | 2,581 (31.4) |
| ARB | 51,818 (25.1) | 2,940 (25.8) | 40,242 (28.3) | 2,499 (30.4) |
| ARNI | 31,400 (15.2) | 1,891 (16.6) | 1,079 (0.8) | 78 (0.9) |
| Aldosterone antagonist | 72,664 (35.2) | 4,038 (35.5) | 21,716 (15.3) | 1,558 (19.0) |
| Beta blocker | 174,795 (84.6) | 9,921 (87.1) | 86,253 (60.6) | 5,703 (69.4) |
| Digoxin | 21,308 (10.3) | 1,510 (13.3) | 4,154 (2.9) | 419 (5.1) |
| Hydralazine/ISDN | 1,609 (0.8) | 81 (0.7) | 317 (0.2) | 26 (0.3) |
| Ivabradine | 2,066 (1.0) | 80 (0.7) | 175 (0.1) | 5 (0.1) |
| Loop diuretic | 113,606 (55.0) | 6,950 (61.0) | 71,134 (50.0) | 5,076 (61.8) |
| **SGLT2i** | 12,191 (5.9) | 698 (6.1) | 6,762 (4.8) | 372 (4.5) |
| Canagliflozin | 1,881 (0.9) | 136 (1.2) | 1,482 (1.0) | 95 (1.2) |
| Dapagliflozin | 5,025 (2.4) | 204 (1.8) | 2,157 (1.5) | 98 (1.2) |
| Empagliflozin | 5,606 (2.7) | 385 (3.4) | 3,307 (2.3) | 192 (2.3) |
| Ertugliflozin | 34 (0.0) | 1 (0.0) | 30 (0.0) | 0 (0.0) |
| **Abbreviations**: ACEi: Angiotensin converting enzyme inhibitor; ARB: Angiotensin II receptor blocker; ARNI: angiotensin receptor/neprilysin inhibitor; CKD: chronic kidney disease; CVA: cerebrovascular accident; HFrEF: Heart failure with reduced ejection fraction; HFpEF: Heart failure with preserved ejection fraction; ISDN: Isosorbide dinitrate; MI: Myocardial infarction; SGLT2i: Sodium-glucose cotransporter-2 inhibitor; SD: Standard deviation | | | | |
